# Expression Levels of WNT Signaling Pathway Genes During Early Tooth Development

**DOI:** 10.1080/15476278.2023.2212583

**Published:** 2023-05-17

**Authors:** Yuhan Song, Fujie Song, Xuan Xiao, Zhifeng Song, Shangfeng Liu

**Affiliations:** a Department of oral mucosa, Shanghai Stomatological Hospital, Fudan University, Shanghai, P. R. China; b Department of first dental clinic, Shanghai Ninth People’s Hospital, Shanghai Jiao Tong University School of Medicine, College of Stomatology, Shanghai Jiao Tong University; National Center for Stomatology, National Clinical Research Center for Oral Diseases, Shanghai Key Laboratory of Stomatology, Shanghai, China; c Shanghai Key Laboratory of Craniomaxillofacial Development and Diseases, Shanghai Stomatological Hospital, Fudan University, Shanghai, P. R. China

**Keywords:** Early tooth development, expression patterns, RNA-seq, tooth agenesis, Wnt signaling pathway

## Abstract

It is known to all that Wnt signaling pathway plays an important role in the early development of tooth. Our previous research found that Wnt signaling pathway played crucial roles in dental development, and mutations in antagonist of Wnt signaling pathway may lead to the formation of supernumerary teeth. However, the expression pattern of Wnt signaling molecules in early development of tooth, especially genes with stage specificity, remains unclear. Hence, we applied RNA-seq analysis to determine the expression levels of wnt signal molecules at five different stages of rat first molar tooth germ. In addition, after literature review we summarized the function of Wnt signaling molecules during tooth development and the relationship between Wnt signaling molecules variation and tooth agenesis. Our research may have implications for exploring the role of Wnt signaling molecules in different stages of tooth development.

## Introduction

The Wnt signaling pathway plays an important role in many processes of biological development, including embryogenesis, tissue homeostasis and wound repair. Several biological behaviors such as cell proliferation, differentiation, polarization, and apoptosis were regulated by the Wnt signal pathway. The Wnt signaling pathway is a complex system that consist of extracellular secreted glycoproteins (19 Wnt ligands), wnt receptors (10 Frizzled receptors, Frz and 2 LDL receptor related protein, Lrp5/6), cytoplasmic proteins (β-catenin, DVL, APC, AXIN and GSK-3β, etc.) and several Wnt-associated molecules (MSX1, DKK1, KREMEN1, and ANTXR1.^1–3^ According to whether it is dependent on the activation of β-catenin, Wnt signaling pathways can be divided into the canonical signaling pathway and the noncanonical signaling pathway. The Wnt ligands, the Fzl receptors and LRP form a complex that activates the downstream canonical β-catenin pathway. Moreover, Wnt-receptor complexes are mediated and controlled by downstream degradation complexes composed of APC, AXIN and GSK-3β. The degradation complex leads to phosphorylation and ubiquitination-mediated degradation of β-catenin. Once a Wnt ligand binds to a Fzl receptor and LRP protein, the degeneration complex in cytoplasm is inhibited, leading to the accumulation of β-catenin.^4–6^ Accumulated β-catenin translocates to the nucleus, interacts with transcription factors TCF (T-cell factor)/LEF (lymphocyte-enhancer-binding factor), and promotes the transcription of Wnt target genes.^7^ Non-canonical Wnt signaling pathways are further divided into Wnt/planar cell polarity (PCP) signaling, Wnt/Ca2+ Pathway, Wnt RTK Pathway and Wnt Frz Pathway which are involved in the process of polarity and cell motility. 8

Tooth development is a dynamic process that includes the bud, cap and bell stages, root development and tooth eruption.^9^ It is universally known that Wnt signaling pathway plays an important role in tooth development.^10^ The Wnt signaling molecules are spatiotemporally activated in tooth development, thereby implying its essential role in the process of odontogenesis. The mutations of Wnt signaling genes may result in tooth agenesis (TA). The genetic link between TA and the Wnt pathway was first evidenced by the identification of a mutation of the Axin2 gene in an oligodontia family.^10^ Mutation of Wnt10a may lead to odonto-onycho-dermal dysplasia, Hypohidrotic ectodermal dysplasia, Schopf-Schulz-Passarge syndrome and many non-syndromic tooth agenesis. Abnormal expression of Wnt10b is related to oligodontia, microdontia, short tooth roots, dental pulp stones, and taurodontism.^11–13^ Mutation of Lrp6 is also associated with oligodontia, mesiodens, fusion of teeth, odontomas, microdontia, long roots, molars with unseparated roots, and taurodontism. 14,15

Tooth development requires regulation of multiple molecules, which is the result of spatiotemporal expression of these molecules. The expression pattern of a gene or signaling are supposed to include not only the expression level but also the location at continuous stages and the study of gene expression level is an important part of learning gene expression patterns. It is known to all that Wnt signaling pathway plays an important role in tooth development. However, the expression pattern of Wnt signaling molecules in development of tooth germ are still unclear. We expect to gain a holistic understanding of the expression of the wnt signaling pathway during early tooth development and to discover genes that are specifically expressed during early tooth development. Here, we determined the expression levels of wnt signal molecules in each stage of rat first molar tooth germ development by RNA seq analysis and after literature review we summarized the function of Wnt signaling molecules during tooth development and the relationship between Wnt signaling molecules variation and tooth agenesis. Our research may have important implications for exploring the role of Wnt signaling molecules in different stages of tooth development.

## Results

### RNA-seq analysis

**Figure 1.**
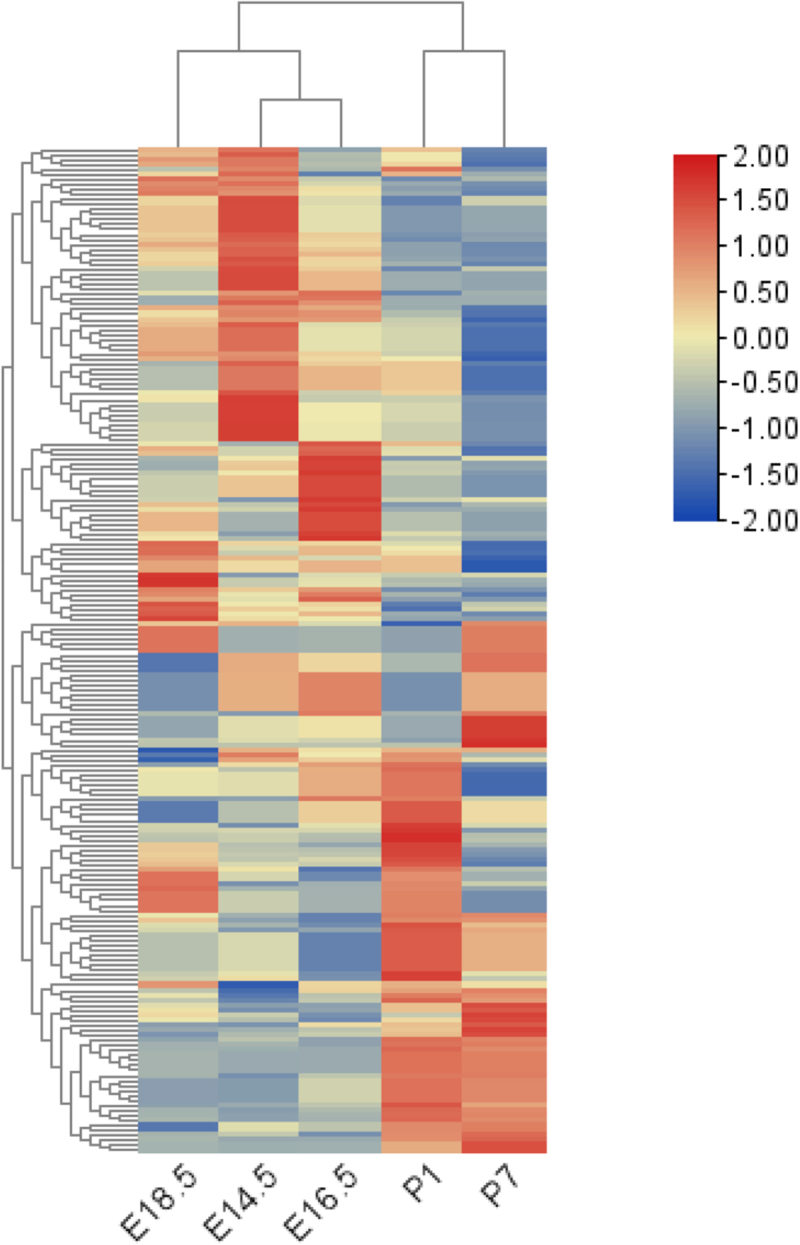
Heatmap shows the relative expression levels of differential genes across different stages of tooth early development (E14.5–P7).

We investigated five time points (E14.5, E16.5, E18.5, P1 and P7) of rat tooth germ development. It is observed that when the embryo developed to E14.5, E16.5 and E18.5, the first molar tooth germ was in bud stage, cap stage and early bell stage respectively, while the tooth germs of 1-and 7-day-old mice were in late bell stage and eruption stage respectively. After sequencing, a total of 143 mRNA related to Wnt signal pathway were detected. Expression of Wnt signaling molecules were displayed in Table 1. Hierarchical clustering analysis revealed that the expression levels of these molecules showed significant time specificity Figure 1. The results showed a high degree of similarity among the tooth germ tissues at embryonic stages, and a low degree of similarity between the embryonic and postnatal tooth germs Figure 1.

**Table 1.** Expression of Wnt signaling molecules during rat tooth germ development.

Gene	E14.5	E16.5	E18.5	P1	P7
Adcy8	0.016	0.081	0.030	0.000	0.000
Adcy9	0.724	1.196	1.303	5.776	4.975
Add1	56.313	41.140	43.719	46.017	32.187
Add2	0.388	0.230	0.248	0.030	0.069
Add3	14.360	16.540	16.516	20.987	19.625
Apc	8.100	8.561	10.913	14.278	12.300
Atf2	17.051	14.203	17.650	12.433	13.722
Bambi	64.547	43.534	82.661	366.512	156.081
Btrc	11.209	9.411	10.330	18.065	16.471
Cacybp	66.993	56.507	53.328	38.559	22.242
Camk2a	0.266	0.383	0.050	0.469	0.000
Camk2b	0.957	1.254	0.842	2.869	2.227
Camk2d	20.199	26.671	22.937	22.122	20.697
Camk2g	24.310	41.241	28.859	25.472	22.382
Ccnd3	73.906	52.275	53.673	25.434	19.872
Cer1	0.553	0.806	2.852	0.316	0.140
Chd8	17.474	18.475	21.148	26.451	13.914
Creb1	12.691	13.775	15.355	8.458	4.297
Csnk1a1	118.607	87.944	90.259	121.792	78.175
Csnk1e	34.616	30.940	34.686	32.465	23.942
Csnk2a1	42.129	33.985	47.836	41.693	63.637
Ctbp1	80.800	53.763	64.548	50.997	32.222
Ctbp2	25.230	21.868	21.743	18.952	18.358
Ctnnb1	413.455	263.582	385.880	320.131	193.689
Ctnnbip1	18.586	11.244	14.660	103.931	11.146
Cul1	66.945	43.481	64.570	35.855	34.552
Cxxc4	4.323	3.407	4.191	2.517	0.517
Daam1	4.469	6.398	5.567	4.615	2.976
Daam2	21.036	36.789	27.931	22.736	9.825
Dact1	7.498	7.140	9.358	4.215	6.387
Dkk1	7.552	4.362	5.262	39.174	5.442
Dkk2	6.198	13.290	4.224	4.682	1.795
Dkk3	12.407	8.222	8.292	30.025	27.688
Dkk4	13.050	10.950	9.055	11.081	7.715
Dvl1	46.062	62.659	47.735	74.318	14.480
Dvl2	24.554	19.259	20.839	16.363	16.952
Dvl3	25.285	29.773	25.406	39.470	37.941
Ep300	14.032	13.941	15.424	17.614	13.128
Fosl1	0.806	1.566	1.011	1.009	1.075
Fzd1	20.989	14.629	8.810	7.114	6.531
Fzd10	10.666	5.326	4.633	4.418	2.007
Fzd2	52.407	29.721	24.961	26.543	14.206
Fzd3	6.168	5.532	8.924	8.622	4.710
Fzd4	1.819	2.304	1.534	1.439	1.241
Fzd5	2.135	2.527	1.712	3.081	2.462
Fzd6	12.920	13.220	15.859	53.238	49.932
Fzd7	0.077	0.515	0.311	0.113	0.039
Fzd8	2.229	1.971	0.944	1.453	2.566
Fzd9	0.722	0.809	0.436	0.450	1.456
Gbp1	0.023	0.023	0.000	0.000	0.000
Gbp2	7.995	5.848	5.820	3.188	3.444
Gbp3	0.034	0.027	0.000	0.033	0.034
Gbp4	0.220	0.332	0.275	0.398	0.119
Gbp5	1.271	0.964	0.564	1.212	0.790
Gpc4	31.489	19.941	28.246	50.848	31.273
Gsk-3β	11.980	10.196	12.913	22.910	21.211
Invs	2.474	2.459	3.949	1.905	0.000
Jun	17.049	13.260	16.128	5.654	19.071
Lrp5	13.174	9.064	14.691	13.702	9.488
Lrp6	13.278	14.962	16.044	13.810	19.741
Map3k7	16.541	17.829	19.218	16.900	13.867
Mapk10	0.238	0.324	0.572	7.831	12.777
Mapk14	29.291	24.578	40.248	28.449	29.146
Mapk8	10.315	10.873	11.099	10.697	7.387
Mapk9	12.347	11.745	9.909	14.452	14.649
Myc	8.971	8.358	11.560	15.352	4.417
Nfatc1	5.567	7.252	7.061	4.689	3.692
Nfatc2	0.496	0.852	0.487	0.838	0.594
Nfatc3	12.177	8.722	10.909	16.309	10.630
Nfatc4	27.927	39.642	18.519	22.084	17.246
Nkd1	13.839	13.420	10.938	8.546	5.460
Nkd2	14.670	22.710	12.204	47.746	85.866
Nlk	7.748	5.541	9.866	17.017	12.540
Notum	8.630	5.898	15.769	26.819	7.288
Pik3ca	9.011	12.143	13.108	12.714	11.986
Pik3cb	1.225	1.680	3.417	3.985	5.799
Pik3cd	3.070	3.011	2.782	11.058	0.000
Pik3cg	0.558	0.850	0.718	0.397	0.378
Plcb1	0.482	0.633	1.597	0.405	0.246
Plcb2	0.219	0.674	0.873	1.430	1.347
Plcb3	15.234	11.342	13.652	14.181	12.188
Plcb4	3.325	3.856	4.305	3.635	2.611
Plcd1	14.715	16.625	10.531	23.731	33.374
Plcd3	5.093	7.756	6.859	14.044	13.901
Plce1	1.748	1.987	1.136	0.441	0.773
Plcg1	71.231	92.843	75.396	74.490	93.367
Plcg2	0.540	0.503	0.464	0.330	1.104
Plch1	1.162	1.122	2.189	4.117	0.187
Plch2	9.434	11.233	12.236	3.655	3.961
Plcl1	1.112	0.835	0.706	0.499	0.680
Plcl2	4.352	2.662	5.075	5.503	3.686
Porcn	8.152	13.251	9.750	10.927	6.938
Ppard	6.749	5.494	7.687	8.118	10.810
Ppp3ca	18.548	22.957	20.454	17.277	18.121
Ppp3cb	42.287	42.055	37.782	30.572	17.677
Ppp3cc	12.282	51.154	16.544	59.909	90.883
Ppp3r1	34.518	26.480	31.538	49.404	52.914
Ppp3r2	0.000	0.000	0.000	0.000	0.261
Prickle1	8.363	8.730	10.231	14.244	12.419
Prickle2	2.271	2.510	2.485	4.382	4.128
Prickle3	6.092	21.432	15.440	25.290	28.133
Prickle4	313.682	253.812	284.599	241.216	107.233
Prkaca	34.750	19.719	23.042	19.972	14.672
Prkca	4.679	3.955	4.744	3.012	2.809
Prkcb	3.631	5.788	1.765	2.371	1.606
Prkcg	18.187	15.037	0.968	18.805	11.926
Psen1	12.582	12.657	13.447	20.832	22.655
Rac1	176.453	86.158	150.045	142.953	68.014
Rac2	20.622	14.785	12.654	4.689	2.633
Rbx1	186.602	300.283	122.306	69.284	42.285
Rhoa	248.423	166.461	209.691	190.984	133.572
Rock2	8.571	11.439	18.397	10.802	11.327
Ror1	6.325	4.882	6.726	3.290	2.455
Ror2	26.932	14.733	23.448	20.330	7.691
Ruvbl1	69.086	42.650	44.662	23.649	11.494
Serpinf1	114.431	64.211	102.738	537.931	515.047
Sfrp5	8.105	4.705	6.184	1.254	0.313
Siah1	15.011	10.920	10.055	15.425	12.780
Skp1	545.003	531.185	430.819	202.943	125.419
Smad3	18.305	13.818	23.791	32.365	31.599
Smad4	26.118	26.182	31.390	35.216	42.754
Sost	0.445	0.612	0.951	0.146	0.000
Sox17	2.757	3.832	2.284	2.089	2.648
Sumo1	201.726	184.457	181.892	99.477	60.460
Tbl1x	25.657	22.071	24.067	20.050	15.948
Wif1	71.632	39.655	65.684	26.883	20.369
Wisp1	5.185	8.644	7.628	25.723	22.845
Wnt10a	13.542	26.080	89.610	81.904	39.079
Wnt10b	8.802	8.385	75.143	64.451	2.657
Wnt11	8.766	5.134	7.138	4.709	1.203
Wnt16	2.673	2.088	0.000	0.000	0.000
Wnt2	0.125	0.148	0.027	0.028	0.126
Wnt2b	0.667	0.604	1.238	0.272	0.247
Wnt3	1.443	0.989	1.745	1.383	0.051
Wnt3a	10.543	3.620	15.694	311.383	258.929
Wnt4	14.618	11.365	13.713	19.420	17.021
Wnt5a	27.869	28.256	43.056	26.524	42.201
Wnt5b	3.537	3.135	2.433	3.024	1.753
Wnt6	51.458	26.080	89.610	81.904	39.079
Wnt7a	1.810	1.068	4.329	5.500	5.283
Wnt7b	6.719	4.644	5.142	3.333	4.496
Wnt8b	0.021	0.059	0.052	0.124	0.055
Wnt9a	2.794	2.983	4.049	1.577	2.396

Since it is difficult to completely distinguish the molecules involved in canonical Wnt signaling pathway and the noncanonical Wnt signaling pathway, therefore we classified and integrated all genes together in Table 1. Canonical Wnt signaling pathway covered 82 different genes, and the noncanonical Wnt signaling pathway covered 81 genes. There were 20 genes with overlap between those two pathways. Besides, expression levels of genes such as Fzd10, Prkaca, Fzd7, Dkk2, Cer1, Wnt10b, Ctnnbip1, Dkk1, Wnt3a, Serpinf1, Dkk3 and Wnt16 showed obvious time specificity.

### Changes in DEG mRNA levels during rat molar development

**Figure 2.**
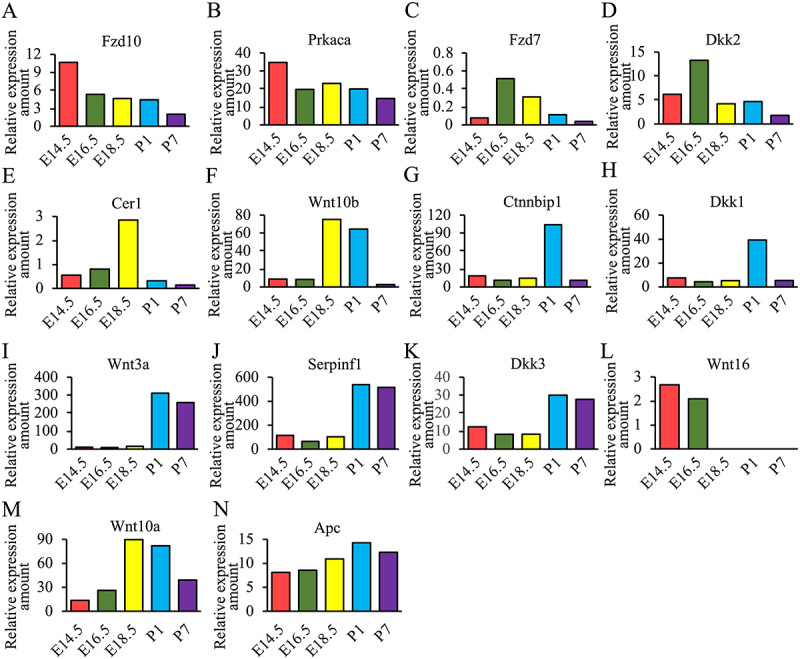
Specific expression of WNT signaling pathway genes in different developmental stages of tooth germs (E14.5–P7).

We summarized the genes with special expression levels in Table 1 and used the relative expression amounts of these genes as the ordinate to draw Figure 2. The mRNA expression levels of Fzd10 and Prkaca were mainly highly expressed mainly in the E14.5 stage. ThemRNAs of Fzd7 and Dkk2 were highly expressed mainly in E16.5 phase, the mRNA ofCer1 was highly expressed in E18.5 phase, while Ctnnbip1 and Dkk1 showed high expression in P1 phase. Wnt10b and Wnt10a were highly expressed in both E18.5and P1 phases. The mRNAs of Wnt3a, Serpinf1 and Dkk3 were highly expressed at the postnatal stage compared to the embryonic stage. In contrast, Wnt16 was expressed only at E14.5 and E16.5 stages. The expression of APC in the five stages of early tooth development is higher than 8.

## Discussion

 We investigated five time points (E14.5, E16.5, E18.5, P1 and P7) of rat tooth germ development. Considering the small size of rat tooth germs, it is difficult to separate dental papilla and dental sac, so we took the whole tooth germs for sequencing. The sequencing results include both epithelial genes and dental papilla genes. Totally 128 mRNA related to Wnt signal pathway of rat tooth germ samples from different stages of development were detected. We observed that the expression levels of these molecules showed significant time specificity, including Fzd10, Prkaca, Fzd7, Dkk2, Cer1, Ctnnbip1, Dkk1, Wnt10b, Wnt3a, Serpinf1, Dkk3, Wnt16. Wnt10b, Dkk1 and Serpinf1 have been found to be associated with tooth agenesis, but little research has been done on the relationship between the other genes mentioned above and tooth germ development. We obtained a relatively complete gene expression profile related to Wnt signaling pathway through RNA sequencing. Our study enriches the understanding of Wnt signal pathways and provides a lot of useful information for subsequent research.

### Wnt signaling molecules and tooth agenesis

Gene mutation may disrupt specific signaling networks and cause a wide variety of selective tooth agenesis patterns. Tooth agenesis can either occur as an isolated condition (non-syndromic tooth agenesis) or can occur as part of a genetic syndrome (syndromic tooth agenesis). Some genes are responsible for syndromic tooth agenesis, including Robinow syndrome and Gardner syndrome. The mutations of APC may cause Gardner syndrome, besides gene mutations of Ror2, Dvl1, Dvl3 and Wnt5a are associated with Robinow syndrome. Genes responsible for tooth agenesis were displayed in Table 2. By searching for HGMD, we found that there are as many as 2037 different mutation forms in APC (Table 2).

**Table 2.** Wnt signaling molecules and tooth agenesis.

Gene	Mutations	OMIM	Syndrome	Dental phenotypes
Apc	2037	175100	Gardner syndrome	Supernumerary teeth and unerupted teeth ^16,17^
Lrp5	157	603506	Non-syndromic tooth agenesis	Dentinogenesis imperfecta,^18^ root malformation ^19^
Wnt10a	20	606268	Hypohidrotic ectodermal dysplasia	Hypodontia, oligodontia, anodontia ^21–23^
			Schopf-Schulz-Passarge syndrome	Conical primary teeth and agenesis of permanent teeth,hypodontia ^24,25^
			Odonto-onycho-dermal dysplasia	Anodontia of permanent teeth ^26^
			Non-syndromic tooth agenesis	Oligodontia,^12, 21, 27–38^ agenesis of the maxillary permanent canines ^39^
Ror2	40	602337	Robinow syndrome	Gingival hyperplasia ^41^
Axin2	42	604025	Non-syndromic tooth agenesis	Oligodontia, agenesis of mandibular and second-premolar ^32, 43–45^
Ctnnb1	43	116806	Non-syndromic tooth agenesis	Tooth agenesis ^47^
Lrp6	21	603507	Non-syndromic tooth agenesis	Oligodontia ^14, 27, 32, 48–52^ mesiodens, fusion of teeth, odontomas, microdontia, long roots, molars with unseparated roots, and taurodontism 15
Serpinf1	26	172860	Osteogenesis imperfecta	Dentinogenesis imperfect ^46^
Sost	15	605740	Craniodiaphyseal dysplasia, van Buchem disease	Tooth abnormalities ^53^
Plcb4	13	600810	Auriculocondylar syndrome	Mandibular condyle hypoplasia ^54^
Dvl1	11	601365	Robinow syndrome	Abnormal alveolar dysplasia ^55^
Wnt7a	7	601570	Al-Awadi-Raas-Rothschild syndrome	Tooth agenesis, microdontia, and taurodontism ^20^
Wnt10b	7	601906	Non-syndromic tooth agenesis	Oligodontia,microdontia, short tooth roots, dental pulp stones, and taurodontism ^11–13^
Dvl3	6	268310	Robinow syndrome	Gingival hyperplasia, dental anomalies ^56^
Wnt5a	5	164975	Robinow syndrome	Misalignment of permanent teeth, gingival hyperplasia ^57,58^
Kremen1	4	609898	Ectodermal dysplasia	Oligodontia ^59^
Dkk1	2	605189	Non-syndromic tooth agenesis	Oligodontia ^27^
Ctbp1	1	602618	Non-syndromic tooth agenesis	Tooth enamel defects ^60^

### Apc

Adenomatous polyposis coli (APC) gene encodes a tumor suppressor protein that acts as an antagonist of the Wnt signaling pathway. It is also involved in cell migration and adhesion, transcriptional activation, and apoptosis.^61^ Mutations in Apc may cause familial adenomatous polyposis (FAP), an autosomal dominant pre-malignant disease that usually progresses to malignancy.^62^ Besides, other diseases such as Gardner syndrome, which is characterized by the presence of multiple intracolonic polyps and extracolonic tumors, can also be caused by Apc mutation. Our previous study found that patients with Gardner syndrome also had multiple impacted and supernumerary teeth.^16^ We found that Apc was expressed at E14.5-P7 and there are up to 2037 different mutation forms in Apc, suggesting that Apc played an important role in all stages of early tooth development.

### Wnt10b

Wingless-Type MMTVIntegration Site Family, Member 10B (Wnt10b) is a ligand of Wnt signaling pathway and has been implicated in regulation of embryogenesis. Multiple variants of Wnt10b were found by mutational analysis in patients with non-syndromic tooth agenesis.^11–13^ Besides, mutations of Wnt10b are associated not only with oligodontia and tooth agenesis, but also with microdontia, short tooth roots, dental pulp stones, and taurodontism.^11^ The mutations of Wnt10b decreased Wnt signaling in dental pulp stem cells, affecting the development of permanent dentition, particularly the lateral incisors.^13^ Wnt10b mutation allele-transfected into stem cells from human exfoliated deciduous teeth resulted in decreased gene transcription, while Wnt10b protein increased with the increase of mutant alleles, indicating that Wnt10b variants have functional effects on gene regulation and reveal that these variants may affect tooth development leading to tooth agenesis.^63^ A. Nadiri et al. observed that the relative amount of Wnt10b was maximal at E14 stage during the development of rat molar tooth germs by western blotting.^64^ At the late bell stage, Wnt10b was mainly detected in the inner dental epithelium, indicating that Wnt10b may be involved in cell proliferation.^64^ Unlike other studies, our study found that Wnt10b was significantly expressed at E18.5 (early bell stage) and P1 (late bell stage) stages. Therefore, the expression of Wnt10b during the tooth germ development needs to be further verified and explored.

### Wnt10a

Wingless-Type MMTV Integration Site Family, Member 10A (Wnt10a) is another important ligand of Wnt signaling pathway, which appears to have specific relevance to skin, its appendages and teeth. Wnt10a is associated with both syndromic and non-syndromic tooth agenesis. Schöpf-Schulz-Passarge syndrome (SSPS) is an autosomal recessive form of ectodermal dysplasia resulting from mutations in Wnt10a.^65^ Tooth agenesis in SSPS patients can be characterized by conical primary teeth, agenesis of permanent teeth and hypodontia.^24^ Odonto-onycho-dermal dysplasia (OODD) is a rare autosomal recessive syndrome mainly characterized by dry hair, anodontia of permanent teeth, smooth tongue, keratoderma, and hyperhidrosis.^26^ Recently, Wnt10a mutations were found in patients with nonsyndromic tooth agenesis as well, mainly characterized by oligodontia and agenesis of the maxillary permanent canines. Upper and lower premolars were the most affected missing teeth.^12,21^ In our research, Wnt10a was significantly expressed in E18.5 (early bell stage) and P1 (late bell stage) stages and the expression level decreased in P7 phase. Our results implied that Wnt10a played an important role in the formation of tooth hard tissue.

### Dkk1

Dickkopf-related protein 1(Dkk1) is a typical antagonist of Wnt/β-catenin signaling by competing for the Wnt receptor LRP5/6. The mutation of Dkk1 may cause oligodontia and short root anomaly.^27,66^ Researchers observed that strong expression of Dkk1 was localized in preodontoblasts on the labial side of the incisors.^67^ At postnatal day 2, Dkk1 is prominently expressed in the preodontoblasts and odontoblasts in mouse molar germs.^68^ In Dkk1 transgenic mice, overexpression of Dkk1 in pulp and odontoblast cells delayed the maturation of dentinogenesis during post-natal development.^69^ The dental crown begins to form in the late bell stage (P1). In this stage, peripheral cells of the dental papilla differentiate into odontoblasts that secrete dentin. Our results demonstrated that Dkk1 was significantly expressed in the P1 phase, which indicated that Dkk1 played an important role in the formation of dentin.

### Serpinf1

Serpin family F member 1 (Serpinf1) encodes the 50-kDa secreted glycoprotein pigment epithelium-derived factor (PEDF), which inhibits Wnt/β-catenin signaling pathway. Mutation of Serpinf1 may cause a unique autosomal recessive disease, Osteogenesis imperfecta (OI) type VI (MIM #610968). OI is characterized by recurrent fractures, progressive skeletal deformities, and growth deficiency, blue or gray discoloration of the sclera and dentinogenesis imperfecta.^70^  Mutations in Serpinf1 have been reported in Chinese individuals with OI.^46^  Our findingSerpinf1 mainly high expressed during postnatal stages, indicated that Serpinf1 played an important role in the formation and mineralization of tooth hard tissues.

### Other important genes

Fzd10 is one of the FZD family receptors which act through canonical Wnt signaling.^42^ Fzd10regulates cell proliferation and mediates Wnt1-induced neurogenesis in the developing spinal cord.^71^ Investigations have demonstrated that mutations in the gene of Prkaca result in the development of adrenocortical adenomas associated with Cushing’s syndrome.^72^ Fzd7 plays a significant role in the regulation of multipotentiality of human pluripotent stem cells and its down-regulation accompanies differentiation and exit from the pluripotent stem cell state Dkk2 plays an important role in ovarian cancer.^73,74^ It has been well documented that Cer1 plays dual roles in neural induction and suppression of mesodermal or endodermal lineages.^40^ We observed that the mRNA expression levels of Fzd10 and Prkaca were mainly high during bud stage (E14.5), Fzd7 and Dkk2 were mainly highly expressed during cap stage (E16.5), and Cer1 was high during early bell stage (E18.5), indicating that they may play important roles in the early stage of tooth germ development. The expression level of Ctnnnbip1 was mainly high during P1 stage (late bell stage), enlightening that Ctnnbipmay be related to the formation and mineralization of enamel and dentin. Compared to the embryonic stages, the mRNA expression levels of Wnt3a and Dkk3 were highly expressed during postnatal stages, which implied that once significant high expression of Wnt3a and Dkk3 were found, it indicated that tooth germ has developed to the stage of hard tissue formation and mineralization. By contrary, Wnt16 was expressed only during E14.5 and E16.5, suggesting that Wnt16 was one of the important markers of early development of tooth germ.

In summary, we determined the expression levels of Wnt signaling molecules at each stage of rat first molar tooth germ development by RNA-seq analysis and after literature review we summarized the relationship between Wnt signaling molecules variants and tooth agenesis. Our data provided important information about novel genes in tooth germ development. Our findings may help to elucidate the molecular mechanisms of tooth germ development and provide a theoretical basis for further studies on the expression and function of genes involved in human tooth development and regeneration.

## Materials and Methods

### Tissue acquisition and preparation

Specific pathogen-free (SPF)-grade Sprague Dawley (SD) rats (Department of Laboratory Animal Science, Fudan University, Shanghai, China) were used in our study. The day when the vaginal plug was formed as a reference of embryonic day 0 (E0), embryos at E14.5, E16.5 and E18.5 stages were used. One-day-old (P1) and seven-day-old (P7) rats were used as well. We separated the embryos from the uterus of pregnant female rats. Each group has 8 mouse embryos, and the sex of the embryos was randomized to both males and females. The first mandibular molar tooth germs were isolated from these embryos and rats using a zoom stereo microscope (Olympus SZ51, Tokyo, Japan) and prepared for subsequent experiments. All molars were dissected by one same researcher. The rats were euthanized with phenobarbital sodium (i.p., 50 mg/kg) anesthesia. All animal experiments were approved by the Institutional Animal Care and Use Committee of Fudan University.

### RNA-seq

Total RNA was obtained from the tooth germ cells in the embryonic (E14.5, E16.5, and E18.5) and postnatal (P1 and P7) phases using TRI reagent (Invitrogen, Carlsbad, CA, USA). More than eight tooth germs were used in each phase. An RNA-seq library was constructed following the manufacturer’s instructions. Then, next-generation sequencing was performed using an Illumina NovaSeq platform (IGA, Udine, Italy). mRNAs related to wnt signal pathway were selected and hierarchical clustering analysis was performed.

### Database search and literature review

We queried the Human Gene Mutation Database(HGMD_®_) (https://www.hgmd.cf.ac.uk/ac/index.php) for all variants of Wnt signaling genes. A comprehensive review of the PubMed database was undertaken for reports in the English language from 1January 2012 to 1 October 2022. The following search terms were used: (tooth [MeSHTerms]) AND (((Wnt Pathway [MeSH Terms]) OR Wnt Pathway, Canonical) OR Wntbeta-Catenin Signaling Pathway). Titles and abstracts were reviewed, and references and citations of relevant studies were also examined.
